# Anomaly detection in Bitcoin market via price return analysis

**DOI:** 10.1371/journal.pone.0218341

**Published:** 2019-06-20

**Authors:** Fa-Bin Shi, Xiao-Qian Sun, Jin-Hua Gao, Li Xu, Hua-Wei Shen, Xue-Qi Cheng

**Affiliations:** 1 CAS Key Laboratory of Network Data Science and Technology, Institute of Computing Technology, Chinese Academy of Sciences, Beijing, China; 2 University of Chinese Academy of Sciences, Beijing, China; Universidad Veracruzana, MEXICO

## Abstract

The Bitcoin market becomes the focus of the economic market since its birth, and it has attracted wide attention from both academia and industry. Due to the absence of regulations in the Bitcoin market, it may be easier to bring some kinds of illegal behaviors. Thus, it raises an interesting question: Is there abnormity or illegal behavior in Bitcoin platforms? To answer this question, we investigate the abnormity in five leading Bitcoin platforms. By analyzing the financial index, *i*.*e*. the normalized logarithmic price return, we find that the properties of price return in bitFlyer are completely different from others. To find the possible reasons, we find that the abnormal ask price and bid price appear simultaneously in bitFlyer, which may be potentially linked to either price manipulation or money laundering. It verifies our conjecture that there may be abnormity or price manipulation in Bitcoin platforms. Furthermore, our findings in price return could also provide an innovative and effective method to detect the abnormity in Bitcoin platforms.

## 1 Introduction

In the last decade, we have witnessed significant changes in finance, impacting both the academic community and financial profession [1–11]. In 2008, a digital currency called Bitcoin was introduced by Nakamoto [12], and it could be sent from users to users in a peer-to-peer Bitcoin network without intermediaries. Due to the opportunities and potential values it presents, it has received extensive attention from all over the world [13, 14], especially from USA, China, and Japan. At 17 : 00 on October 10, 2018, the price of a Bitcoin reaches 6,558.46 dollars; the 24-hour trading volume of Bitcoin is more than three billion dollars, which is 37 times greater than the daily trading volume of General Electric Company; and the total market capitalization of Bitcoin is more than one hundred billion dollars, which is approximately equal to the market capitalization of General Electric Company.

Unlike the traditional currencies, Bitcoin is the first decentralized digital currency without a central bank or single administrator [15]. As a new kind of digital currency, Bitcoin can be traded online at any time and exchanged on hundreds of Bitcoin platforms in many countries [16], while traders in traditional financial markets buy or sell the specific share only in a single platform. The trading rules are developed by the exchange platforms themselves, and these rules exist a variety of vulnerabilities. Moreover, the traders in the Bitcoin market are anonymous and do not have to use real identification to trade. All of these bring enormous challenges for regulation.

Compared to other financial markets, Bitcoin market lacks of strong regulation [17]. It is easier to bring some kinds of illegal behaviors in the Bitcoin market. Thus, it raises an interesting question: Is there abnormity or illegal behavior in Bitcoin platforms? To answer this question, we collect data from several Bitcoin platforms and investigate the properties of the Bitcoin market, especially the price return. The price return is one of the most important properties for financial markets, which is the key to understand and model financial markets, and quantify risk [18, 19]. In previous studies, researchers have found that the distribution of price return displays a fat tail [20–25]. And the price return is absence of linear autocorrelation [26–29], while the absolute value of price return displays a long-range memory [30–34].

Moreover, with the development of Bitcoin market, a number of papers have been dedicated to studying the Bitcoin market. They found that one or two special Bitcoin platforms exhibit similar behaviors with traditional financial markets. Osterrieder *et al*. [35] found that the distribution of price return exhibits not only high volatility but also strong non-normal characteristics and heavy tails. Jiang *et al*. [36] and Lahmiri *et al*. [37] found that the price return in Bitcoin platforms exists a long-range memory. Besides, Bariviera *et al*. [38] studied the stylized fact of the Bitcoin market. However, all of these previous studies are concentrated on the characteristics of the Bitcoin market.

Distinguished from these empirical studies of price return, our work focuses on the abnormity in different Bitcoin platforms which may be caused by illegal activities. In this paper, we investigate the properties of price return in five leading Bitcoin platforms, including OKCoin, BTC-e, Coinbase, bitFlyer, and Bitfinex. We observe that OKCoin, BTC-e, Coinbase, and Bitfinex have similar characteristics of price return, but bitFlyer not. The kurtosis *κ* is larger than 1,000 in bitFlyer when time interval Δ*t* = 2 min, while it is smaller than 50 in the other four platforms. And the power-law exponent *α* for the price return distribution is smaller than 2 when time interval Δ*t* < 10 min, while *α* > 2 for other platforms. Besides, the linear autocorrelation and the autocorrelation of absolute price return decay much more quickly than those of other platforms. To find the possible reasons, we investigate the price of the order, finding that the abnormal ask price and the bid price simultaneously appear in bitFlyer, which is an evidence of abnormal transactions or money laundering. Furthermore, it has been reported that bitFlyer was punished by the Japan Financial Services Agency. Thus, the abnormity in bitFlyer may be caused by the price manipulation or money laundering, which verifies our conjecture that there may be abnormity or price manipulation in Bitcoin platforms. And the study in price return could also provide an innovative and effective method to detect the abnormity in Bitcoin platforms.

## 2 Datasets and definitions

In this section, we introduce the preparations before the experiment, including the datasets and the definition of price return.

### 2.1 Dataset description

The increasing growth of Bitcoin exchange offers a rare opportunity to record a large amount of Bitcoin order book data across different countries for a long time. To ensure that the result of the experiment is persuasive and reliable, we choose five leading exchange platforms in China, Russia, United States, Japan as follows:

1. OKCoin is the largest Bitcoin exchange platform in China which was founded in 2013. It consists of millions of users and billions of turnovers per day. From 2016 to 2017, the trading volume of Bitcoin in OKCoin is roughly 39% of the total volume of Bitcoin market [39].
2. Bitfinex is headquartered in Hong Kong and registered in the British Virgin Islands. It has been the largest Bitcoin platform in the world, accounting for 10% of total transactions. Until now, it is still the top 10 Bitcoin platform.
3. BTC-e is a leading exchange headquartered in Russia. It is one of the earliest Bitcoin exchange platforms in the world. Up to February 2015, BTC-e handled around 3% of Bitcoin exchange volume.
4. Coinbase is a Bitcoin trading platform established in the USA. The users in Coinbase reached 20 million in 2018, which is more than other Bitcoin platforms. And it is the first broker-dealer to offer SEC-regulated cryptocurrency securities in the USA.
5. bitFlyer is the most popular Bitcoin exchange platform in Japan [40]. The number of users in bitFlyer is more than 2 million. It was reported that the trading volume ranked the first after the legalization of Bitcoin in Japan.

The datasets collected from these five Bitcoin platforms record the price sequence of the order book. And they are collected every a few seconds during the observation period. Due to the data collection limitation, we collect data from these Bitcoin platforms in different time periods, varying from 2 months to 9 months. The description of these datasets is shown in Table 1.

**Table 1:** The description of the data collected from OKCoin, Bitfinex, BTC-e, Coinbase and bitFlyer.

Platform	Currency	Date	Country	Records
OKCoin	CNY	Mar. 1, 2017—Jul. 28, 2017	China	1.2 × 10^7^
Bitfinex	USD	Mar. 11, 2017—Nov. 7, 2017	USA	2.0 × 10^6^
BTC-e	USD	May. 3, 2017—Jul. 26, 2017	Russia	3.7 × 10^6^
Coinbase	USD	Jan. 23, 2018—Sept. 9, 2018	USA	3.0 × 10^7^
bitFlyer	JPY	May. 16, 2018—Jul. 16, 2018	Japan	6.9 × 10^6^

### 2.2 The definition of price return

The best ask *a*(*t*) (or best bid *b*(*t*)) is defined to be the lowest ask price (or highest bid price) at time *t*. And the midprice is defined as the average of *a*(*t*) and *b*(*t*), *i*.*e*. $p\left(t\right)≔\frac{a(t)+b(t)}{2}$. For a fixed time interval Δ*t*, the logarithmic price return is defined to be

r(t,Δt)≔lnp(t+Δt)-lnp(t). (1)

**Fig 1:**
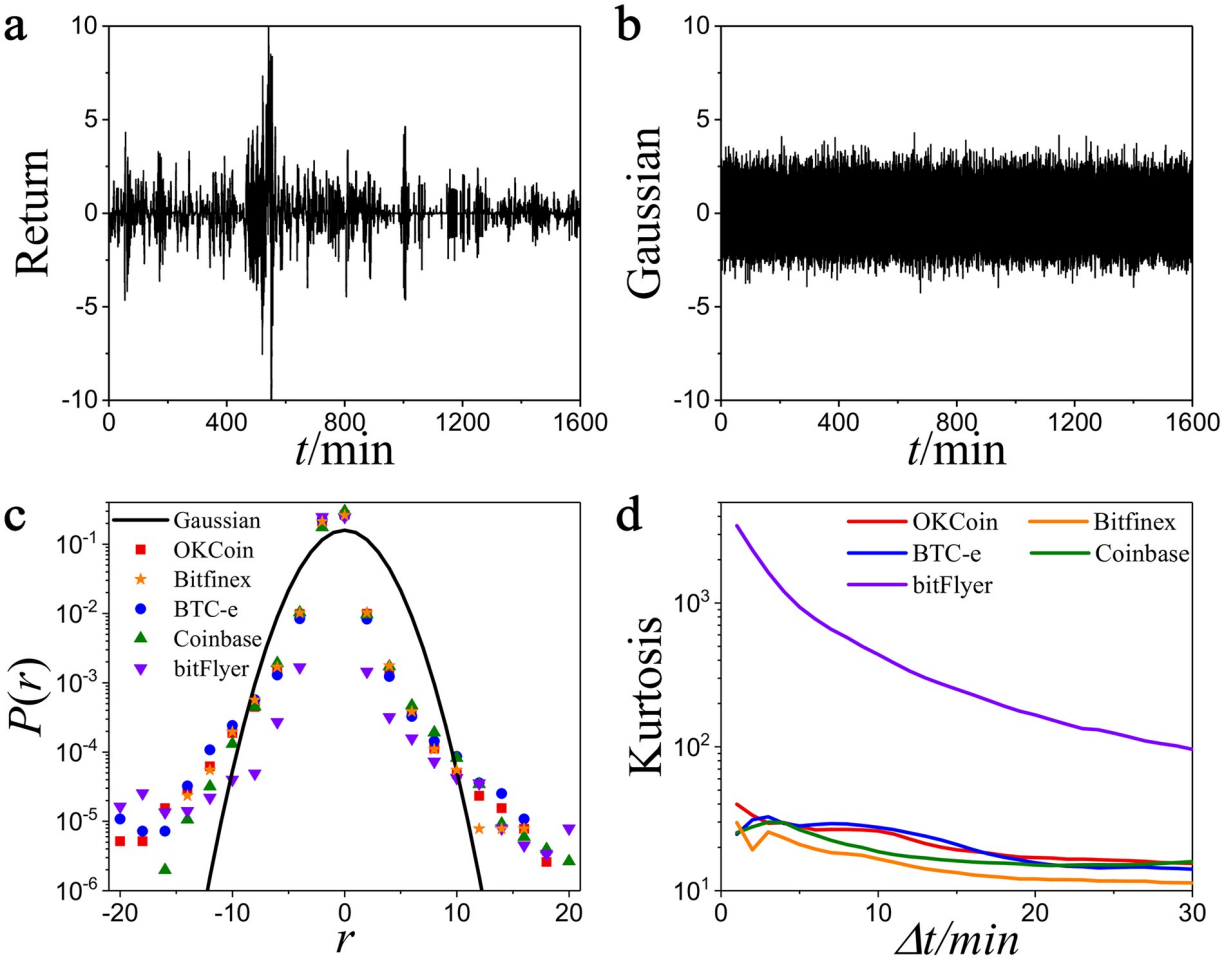
The fat-tail of price return distribution. **a**, An example of normalized price return in OKCoin from 14:04 on March 3, 2017 to 22:45 on March 4, 2017. **b**, An example of noise signal sampled from a Gaussian distribution. **c**, The distribution of normalized price return in different Bitcoin platforms compared with Gaussian distribution when Δ*t* = 2 min. **d**, The kurtosis *κ* versus Δ*t* in different Bitcoin platforms.

As a matter of fact, the normalized logarithmic price return is more frequently adopted in previous works [23–25]. The normalized logarithmic price return is defined as $\frac{r(t,\Delta t)-\langle r(t,\Delta t)\rangle }{\sigma }$, where 〈*r*(*t*, Δ*t*)〉 and *σ* are the mean and standard derivation of *r*(*t*, Δ*t*) over the entire time series, respectively. The price return mentioned in this work is the normalized logarithmic price return. In Fig 1**a**, we plot the normalized logarithmic price return in OKCoin from 14:04 on March 3, 2017 to 22:45 on March 4, 2017. The maximum fluctuation of price return is over 10 times of standard deviation. To get an intuitive understanding of the price return, we compare it with Gaussian noise. We plot the Gaussian noisy signal in Fig 1**b**, where the fluctuations in Gaussian noise range from 0 to 3 times of standard deviation. The fluctuations in price return are much larger than those of the Gaussian noise.

## 3 Results

In this section, we analyze the properties of price return, including the fat-tail of the price return distribution, the power-law decay of price return distribution, and the dependence property of price return. These are three key properties for price return, which have been proven to be common in varieties of financial markets. But it may be different when the market lacks of strong regulation. Therefore we use these properties to verify our conjecture.

### 3.1 The fat-tail of price return distribution

The probability distribution is one of the most important properties for price return. We measure the probability distribution of price return *P*(*r*) in different Bitcoin platforms with Δ*t* = 2 min (Fig 1**c**). We find that the probability distributions of price return in different platforms have similar characteristics. The probability of price return reaches the maximum when |*r*| = 0, and *P*(|*r*|) decreases as the absolute value of price return |*r*| increases. Moreover, the probability of price return in the tail is higher than that of the Gaussian distribution, in agreement with the previous studies in other financial markets [21–25, 28, 29].

Although the distributions of price return in the five Bitcoin platforms are all fat-tail, they have obvious differences in the tail. To distinguish these differences, the kurtosis *κ* is introduced as

$$\kappa ≔\frac{\left\langle {\left(r\left(t,\Delta t\right)-\left\langle r\left(t,\Delta t\right)\right\rangle \right)}^{4}\right\rangle }{\sigma^{4}}-3,$$ (2)

where 〈*r*(*t*, Δ*t*)〉 and *σ* are the mean and the standard derivation of *r*(*t*, Δ*t*), respectively. As a measure of fat-tail of the probability distribution, the kurtosis *κ* > 0 if the distribution is fat-tail, while *κ* = 0 if the distribution is Gaussian distribution. We calculate *κ* with Δ*t* = 2 min (Table 2). It is observed that the kurtosis *κ* > 0 in all five platforms. However, the kurtosis *κ* = 2337.68 in bitFlyer, while *κ* is smaller than 50 in other four platforms. The kurtosis *κ* in bitFlyer is pretty higher than other Bitcoin platforms.

**Table 2:** The statistical information about normalized price return when Δ*t* = 2 min in different Bitcoin platforms.

Platform	Kurtosis	*α*	*α*^+^	*α*^−^
OKCoin	33.45	3.34	3.14	3.36
Bitfinex	19.22	3.23	3.17	2.94
BTC-e	31.14	2.61	2.94	2.87
Coinbase	27.82	3.33	3.17	3.92
bitFlyer	**2337.68**	**1.45**	**1.48**	**1.46**

To confirm whether this difference between bitFlyer and other platforms is universal for different time intervals, we investigate the properties of price return distribution over different time interval Δ*t*. Fig 1**d** shows that the kurtosis *κ* is bigger than 0 for different Δ*t*. The kurtosis *κ* decreases as Δ*t* increases, implying that the distribution of price return is no longer heavy as Δ*t* increases. But the kurtosis *κ* in bitFlyer is significantly larger than that in other platforms when Δ*t* < 30 min. It implies that the number of extreme values in bitFlyer is more than that of other platforms. The risk in the financial markets is often governed by unpredictable extreme return, so the risk in bitFlyer is higher than the risk in other Bitcoin platforms. In this aspect, bitFlyer seems to be more abnormal compared to other four Bitcoin platforms.

### 3.2 The decay of price return distribution

**Fig 2:**
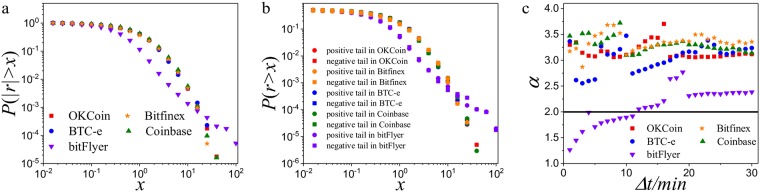
The decay of price return distribution. **a**, The cumulative distribution of normalized absolute price return in log-log plot. **b**, The cumulative distribution of normalized price return in log-log plot. **c**, The power-law exponent *α* versus Δ*t* in different Bitcoin platforms.

We investigate the decay of the price return. In previous studies [28, 29], it was reported that the cumulative distribution of price return *P*(|*r*| > *x*) follows a power-law decay. Fig 2**a** shows that the tail of distribution decays with power-law *P*(|*r*| > *x*) ∼ *x*^−*α*^ in Bitcoin platforms for some *α* when Δ*t* = 2 min. In addition, we find that the positive tail and negative tail are both approximately power-law decay (Fig 2**b**). But the power-law exponents *α* vary in different Bitcoin platforms (Table 2). Both positive tail exponent *α*^+^ and negative tail exponent *α*^−^ in OKCoin, Bitfinex, BTC-e, and Coinbase are more than 2, showing that the price return has finite variance. However, the power-law exponent *α* ≈ 1.45, *α*^+^ ≈ 1.48 and *α*^−^ ≈ 1.46 are all less than 2 in bitFlyer.

Moreover, we investigate the power-law exponent *α* of price return distribution in different time intervals. The power-law exponent *α* ranges from 2.7 to 3.7 in OKCoin, BTC-e, Coinbase, and Bitfinex (Fig 2**c**). However, the exponent *α* in bitFlyer increases as Δ*t* increases. When Δ*t* < 10 min, *α* is smaller than 2. Up to now, no markets have been reported to own a small *α* < 2. When the exponent *α* < 2, it suggests that the price return in this financial market has infinite variance. However, in traditional financial markets, most of the trading behaviors are under strict regulation. The fluctuations of price are relatively small, resulting in the fact that *α* > 2. The unique property of *α* < 2 in bitFlyer indicates that the risk in bitFlyer is much larger than other Bitcoin platforms and other financial markets.

### 3.3 The dependence property of price return

We investigate the dependence property of price return via the analysis on the autocorrelation of the price return. We first conduct the Ljung-Box test in price return series and absolute price return series. The results reject the null hypothesis, indicating that there exists autocorrelation in price return.

**Fig 3:**
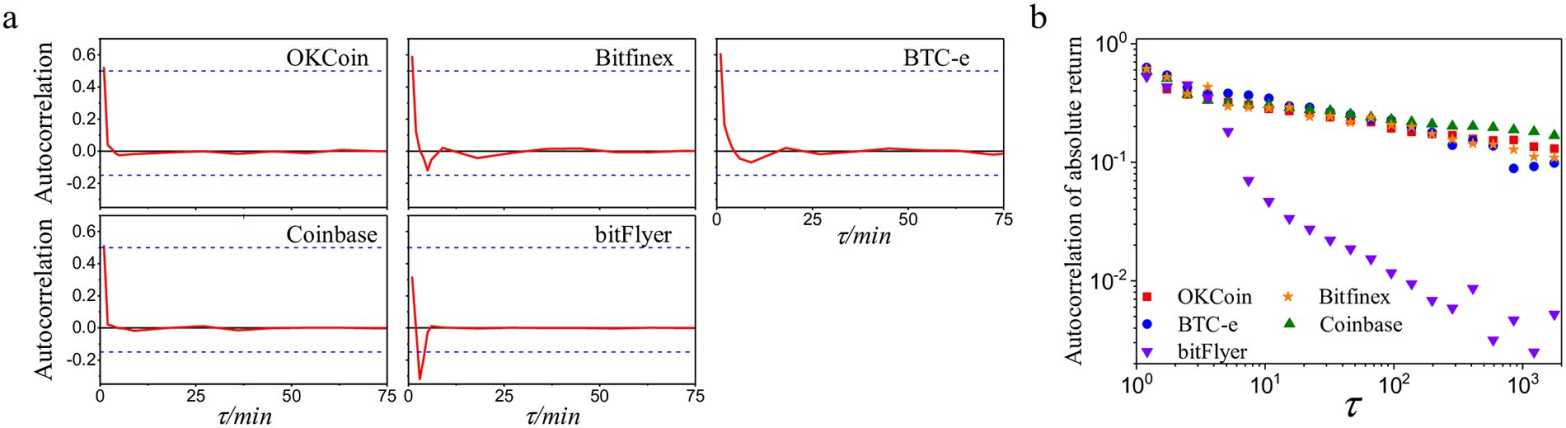
The dependence property of price return. **a**, The linear autocorrelation of normalized price return in different Bitcoin platforms when time interval Δ*t* = 2 min. **b**, The autocorrelation of normalized absolute price return in different Bitcoin platforms when time interval Δ*t* = 2 min.

Next, we calculate the linear autocorrelation given time interval Δ*t*. The linear autocorrelation can be calculated to be

$$cor\left(r\left(t,\Delta t\right),r\left(t+\tau ,\Delta t\right)\right)=\frac{\left\langle (r(t,\Delta t)-\langle r(t,\Delta t)\rangle )(r(t+\tau ,\Delta t)-\langle r(t,\Delta t)\rangle )\right\rangle }{\sigma^{2}},$$ (3)

where 〈*r*(*t*, Δ*t*)〉 and *σ* are the mean and the standard derivation of *r*(*t*, Δ*t*), respectively. In Fig 3**a**, we plot the autocorrelation of price return in different Bitcoin platforms. It shows that the autocorrelation *cor*(*r*(*t*, Δ*t*), *r*(*t* + *τ*, Δ*t*)) > 0 given Δ*t* = 2 min when *τ* is small. But it decreases from positive to negative as *τ* grows. Finally, the autocorrelation falls to 0 as *τ* grows, and it can be considered as irrelevance when *τ* > 20 min. However, there exist differences in autocorrelation between bitFlyer and other Bitcoin platforms. The maximum positive autocorrelation is greater than 0.5 and the minimum negative autocorrelation is smaller than -0.15 in OKCoin, BTC-e, Coinbase, and Bitfinex. In bitFlyer, the maximum positive autocorrelation is roughly 0.35 and the minimum negative autocorrelation is below -0.3. Thus, compared to other Bitcoin platforms, bitFlyer has weaker positive autocorrelation and stronger negative autocorrelation. The weak positive autocorrelation indicates that bitFlyer appears more fluctuations. The strong negative autocorrelation is brought about by the market prevention that protects Bitcoin price from deviating actual value, implying that there are more violent fluctuations in bitFlyer.

Furthermore, we study the nonlinear dependence of price return. We plot the autocorrelation of absolute return *Cor*(|*r*(*t*, Δ*t*)|, |*r*(*t* + *τ*, Δ*t*)|) as shown in Fig 3**b** when Δ*t* = 2 min. The autocorrelation of absolute value of price return decreases as *τ* increases. It decays more slowly compared with the linear autocorrelation. The autocorrelation *Cor*(|*r*(*t*, Δ*t*)|, |*r*(*t* + *τ*, Δ*t*)|) ≈ 0.2 when *τ* = 1000 min in OKCoin, BTC-e, Coinbase, and Bitfinex, showing a long memory in the absolute value of price return |*r*(*t*)|. This phenomenon is called as “volatility clustering” in previous studies [22, 24, 25], commonly across a wide range of financial markets. But it is not significant in bitFlyer. The autocorrelation *Cor*(|*r*(*t*, Δ*t*)|, |*r*(*t* + *τ*, Δ*t*)|) ≈ 0.05 when *τ* = 10 min; and the autocorrelation *Cor*(|*r*(*t*, Δ*t*)|, |*r*(*t* + *τ*, Δ*t*)|) ≈ 0.01 when *τ* = 100 min, smaller than other Bitcoin platforms. The autocorrelation of absolute return decreases more quickly, implying that the fluctuations in bitFlyer are more random and unpredictable.

### 3.4 The possible reasons of abnormity

In previous sections, we find that the properties of price return in different Bitcoin platforms are incompletely consistent. The phenomena that have been largely observed in financial markets, such as fat-tail of price return, the absence of autocorrelation, and volatility clustering, are also found in the Bitcoin market. However, it can be shown that the properties are fairly different in bitFlyer from other Bitcoin platforms. The kurtosis *κ* of the price return distribution, the power-law exponent *α* of the price return distribution, and the autocorrelation of price return in bitFlyer all highly deviate from other platforms.

In spite of the different data coverage, all markets except bitFlyer exhibit similar statistical behaviors, but bitFlyer stands apart. In fact, the phenomena, such as fat-tail of price return, the absence of autocorrelation, and the volatility clustering, are well known as the stylized facts, which have been proven to be common across a wide range of instruments, markets, and time periods [22, 28, 29]. It indicates that these behaviors are independent of the selection of time periods. In this work, the time periods in OKCoin, Bitfinex, BTC-e, and Coinbase are different and time length varies from 2 months to 9 months, but the statistical behaviors are consistent and the same as many previous studies [21–25, 28, 29]. Thus, the obvious difference in bitFlyer from other platforms may be caused by possible illegal actions.

To find the possible reasons of abnormity, we consider the best price that traders quote. It is because the price adopted in this paper is the average of the best ask price and the best bid price. Thus, the abnormity in price return may be caused by the abnormity in best price that traders quote. We introduce the ratio: $ratio^{b}\left(t\right)≔\frac{b_{1}\left(t\right)-b_{2}\left(t\right)}{b_{2}\left(t\right)}$ and $ratio^{a}\left(t\right)≔\frac{a_{1}\left(t\right)-a_{2}\left(t\right)}{a_{2}\left(t\right)}$, where *b*_1_(*t*)(*or* *a*_1_(*t*)), *b*_2_(*t*)(*or* *a*_2_(*t*)) are the best bid (or ask) price and second best bid (or ask) price respectively. The value of *ratio*^*b*^(or *ratio*^*a*^) evaluate the deviation from best bid (or ask) order to second bid (or ask) order.

**Fig 4:**
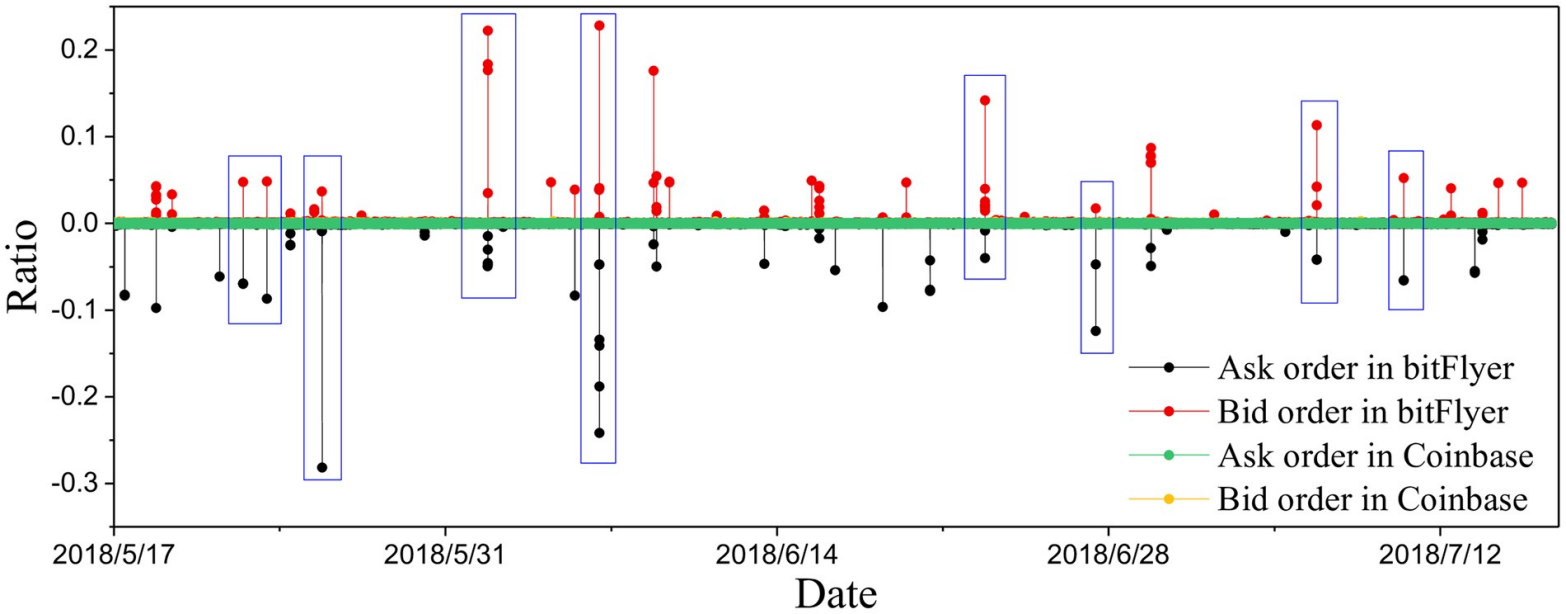
The values *ratio*^*a*^ and *ratio*^*b*^ in bitFlyer and Coinbase, sampled from May 17, 2018 to July 16, 2018.

In Fig 4, we plot *ratio*^*a*^ and *ratio*^*b*^ in bitFlyer and Coinbase using the data collected from May 17, 2018 to July 16, 2018. It is found that most of *ratio*^*a*^ and *ratio*^*b*^ are smaller than 0.01, while some *ratio*^*a*^ and *ratio*^*b*^ in bitFlyer are fairly larger than 0.01. In addition, the extreme values of *ratio*^*a*^ and *ratio*^*b*^ appear simultaneously. It means that buyers and sellers quote price far beyond current best price (higher than best bid or lower than best ask) at the same time. In normal financial markets, it rarely occurs that the abnormal values of *ratio*^*a*^ and *ratio*^*b*^ frequently appear at the same time. One possible explanation is that one trader places abnormal ask orders (lower than best ask) while another trader places abnormal bid orders (higher than best bid) simultaneously. They try to manipulate the price by creating a false impression of an active market. If the abnormal ask orders and the abnormal bid orders are placed concurrently again and again during a certain time period, it may not be coincidental but deliberate. Thus, it may be potentially linked to either price manipulation or money laundering.

Furthermore, bitFlyer was punished by the Japan Financial Services Agency due to the Know Your Customer (KYC) policy vulnerability on June 22, 2018. The KYC process verifies the identity of its clients and assess potential risks of illegal intentions for the business relationship. It prevents financial institutions from being used, intentionally or unintentionally, by criminal elements for money laundering activities. The vulnerability of KYC policy may result in the money laundering or price manipulation, which verifies our conjecture.

Therefore, the abnormity in bitFlyer may be caused by price manipulation or money laundering, essentially due to the absence of strict regulation. To achieve price manipulation or money laundering, the ask (bid) price that traders quote is abnormal, naturally lower (higher) than current ask (bid) price. Thus the kurtosis *κ* is quite large and the power-law exponent *α* of price return distribution is relatively small. And these manipulating behaviors which are different from normal trading behaviors will reduce linear autocorrelation and nonlinear autocorrelation.

## 4 Conclusion

In this paper, we investigate the properties of price return in five leading Bitcoin platforms, including OKCoin, BTC-e, Coinbase, bitFlyer, and Bitfinex. We find that the price returns in OKCoin, BTC-e, Coinbase, and Bitfinex have similar characteristics, including the fat-tail of the price return distribution, the power-law decay of price return distribution, and the autocorrelations of price return, which are entirely different in bitFlyer.

We analyze the price of the bid/ask order, finding that the abnormal ask price and bid price appear in bitFlyer almost simultaneously, which is a characteristic of abnormal transactions. Moreover, bitFlyer was punished by the Japan Financial Services Agency due to KYC Policy vulnerability on June 22, 2018. It verifies our conjecture that there may exist abnormity or manipulation in Bitcoin platforms.

In the future, we would collect more information to confirm whether bitFlyer is in price manipulation or money laundering. Besides, we would validate our conclusion in more Bitcoin exchange platforms.

## References

[1] Bariviera AF, Martín MT, Plastino A, Vampa V. LIBOR troubles: Anomalous movements detection based on maximum entropy. Physica A: Statistical Mechanics and its Applications. 2016;449:401–407. 10.1016/j.physa.2016.01.005

[2] Bariviera AF, Guercio MB, Martinez LB, Rosso OA. A permutation information theory tour through different interest rate maturities: the Libor case. Philosophical Transactions of the Royal Society A: Mathematical, Physical and Engineering Sciences. 2015;373(2056):20150119 10.1098/rsta.2015.0119 26527817

[3] Mantegna RN, Stanley HE. Scaling behaviour in the dynamics of an economic index. Nature. 1995;376(6535):46 10.1038/376046a0

[4] Gabaix X, Gopikrishnan P, Plerou V, Stanley HE. A theory of power-law distributions in financial market fluctuations. Nature. 2003;423(6937):267 10.1038/nature01624 12748636

[5] Plerou V, Gopikrishnan P, Stanley HE. Econophysics: Two-phase behaviour of financial markets. Nature. 2003;421(6919):130 10.1038/421130a 12520293

[6] Shi FB, Sun XQ, Shen HW, Cheng XQ. Detect colluded stock manipulation via clique in trading network. Physica A: Statistical Mechanics and its Applications. 2019;513:565–571. 10.1016/j.physa.2018.09.011

[7] Sun XQ, Cheng XQ, Shen HW, Wang ZY. Distinguishing manipulated stocks via trading network analysis. Physica A: Statistical Mechanics and its Applications. 2011;390(20):3427–3434. 10.1016/j.physa.2011.04.006

[8] Sun XQ, Shen HW, Cheng XQ, Wang ZY. Degree-strength correlation reveals anomalous trading behavior. PLOS ONE. 2012;7(10):e45598 10.1371/journal.pone.0045598 23082114 PMC3474833

[9] Sun XQ, Shen HW, Cheng XQ, Zhang Y. Detecting anomalous traders using multi-slice network analysis. Physica A: Statistical Mechanics and its Applications. 2017;473:1–9. 10.1016/j.physa.2016.12.052

[10] Sun XQ, Shen HW, Cheng XQ. Trading network predicts stock price. Scientific reports. 2014;4:3711 10.1038/srep03711 24429767 PMC5379184

[11] Sun XQ, Shen HW, Cheng XQ, Zhang Y. Market confidence predicts stock price: Beyond supply and demand. PloS one. 2016;11(7):e0158742 10.1371/journal.pone.0158742 27391816 PMC4938583

[12] Nakamoto S. Bitcoin: A peer-to-peer electronic cash system. 2008.

[13] Böhme R, Christin N, Edelman B, Moore T. Bitcoin: Economics, technology, and governance. Journal of Economic Perspectives. 2015;29(2):213–238. 10.1257/jep.29.2.213

[14] Urquhart A. The inefficiency of Bitcoin. Economics Letters. 2016;148:80–82. 10.1016/j.econlet.2016.09.019

[15] Tschorsch F, Scheuermann B. Bitcoin and beyond: A technical survey on decentralized digital currencies. IEEE Communications Surveys & Tutorials. 2016;18(3):2084–2123. 10.1109/COMST.2016.2535718

[16] Brandvold M, Molnár P, Vagstad K, Valstad OCA. Price discovery on Bitcoin exchanges. Journal of International Financial Markets, Institutions and Money. 2015;36:18–35. 10.1016/j.intfin.2015.02.010

[17] Doguet JJ. The Nature of the Form: Legal and Regulatory issues surrounding the Bitcoin digital currency system. Louisiana Law Review. 2013;73(4):9.

[18] Bouchaud JP, Potters M. Theory of financial risk and derivative pricing: from statistical physics to risk management. Cambridge University Press; 2003.

[19] Johnson NF, Jefferies P, Hui PM, et al Financial market complexity. OUP Catalogue. 2003.

[20] Botta F, Moat HS, Stanley HE, Preis T. Quantifying stock return distributions in financial markets. PLOS ONE. 2015;10(9):e0135600 10.1371/journal.pone.0135600 26327593 PMC4556674

[21] Mandelbrot BB. The variation of certain speculative prices In: Fractals and scaling in finance. Springer; 1997 p. 371–418.

[22] Cont R. Empirical properties of asset returns: stylized facts and statistical issues. Quantitative Finance. 2001; 1:2, 223–236. 10.1080/713665670

[23] Plerou V, Stanley HE. Stock return distributions: tests of scaling and universality from three distinct stock markets. Physical Review E. 2008;77(3):037101 10.1103/PhysRevE.77.037101 18517560

[24] Gopikrishnan P, Meyer M, Amaral LN, Stanley HE. Inverse cubic law for the distribution of stock price variations. The European Physical Journal B-Condensed Matter and Complex Systems. 1998;3(2):139–140. 10.1007/s100510050292

[25] Gu GF, Chen W, Zhou WX. Empirical distributions of Chinese stock returns at different microscopic timescales. Physica A: Statistical Mechanics and its Applications. 2008;387(2-3):495–502. 10.1016/j.physa.2007.10.012

[26] Malkiel BG, Fama EF. Efficient capital markets: A review of theory and empirical work. Journal of Finance. 1970;25(2):383–417. 10.2307/2325486

[27] Campbell JY, Lo AW, MacKinlay AC, et al The econometrics of financial markets. vol. 2 Princeton University Press, Princeton, NJ; 1997.

[28] Chakraborti A, Toke IM, Patriarca M, Abergel F. Econophysics review: I. Empirical facts. Quantitative Finance. 2011;11(7):991–1012. 10.1080/14697688.2010.539248

[29] Gould MD, Porter MA, Williams S, McDonald M, Fenn DJ, Howison SD. Limit order books. Quantitative Finance. 2013;13(11):1709–1742. 10.1080/14697688.2013.803148

[30] Liu Y, Cizeau P, Meyer M, Peng CK, Stanley HE. Correlations in economic time series. Physica A: Statistical Mechanics and its Applications. 1997;245(3-4):437–440. 10.1016/S0378-4371(97)00368-3

[31] Stanley HE, Plerou V, Gabaix X. A statistical physics view of financial fluctuations: Evidence for scaling and universality. Physica A: Statistical Mechanics and its Applications. 2008;387(15):3967–3981. 10.1016/j.physa.2008.01.093

[32] Cont R. Long range dependence in financial markets In: Fractals in engineering. Springer; 2005 p. 159–179.

[33] Cont R, Potters M, Bouchaud JP. Scaling in stock market data: stable laws and beyond In: Scale invariance and beyond. Springer; 1997 p. 75–85.

[34] Zheng Z, Qiao Z, Takaishi T, Stanley HE, Li B. Realized volatility and absolute return volatility: a comparison indicating market risk. PLOS ONE. 2014;9(7):e102940 10.1371/journal.pone.0102940 25054439 PMC4108408

[35] Osterrieder J, Lorenz J. A statistical risk assessment of Bitcoin and its extreme tail behavior. Annals of Financial Economics. 2017;12(01):1750003 10.1142/S2010495217500038

[36] Jiang Y, Nie H, Ruan W. Time-varying long-term memory in Bitcoin market. Finance Research Letters. 2018;25:280–284. 10.1016/j.frl.2017.12.009

[37] Lahmiri S, Bekiros S, Salvi A. Long-range memory, distributional variation and randomness of bitcoin volatility. Chaos, Solitons & Fractals. 2018;107:43–48. 10.1016/j.chaos.2017.12.018

[38] Bariviera AF, Basgall MJ, Hasperué W, Naiouf M. Some stylized facts of the Bitcoin market. Physica A: Statistical Mechanics and its Applications. 2017;484:82–90. 10.1016/j.physa.2017.04.159

[39] Guo T, Antulov-Fantulin N. Predicting short-term bitcoin price fluctuations from buy and sell orders. arXiv preprint arXiv:180204065. 2018.

[40] Makarov I, Schoar A. Trading and Arbitrage in Cryptocurrency Markets. 2018.

